# Individualistic impact of unit operations of production, at household level, on some antinutritional factors in selected cowpea‐based food products

**DOI:** 10.1002/fsn3.306

**Published:** 2015-11-01

**Authors:** Mathew K. Bolade

**Affiliations:** ^1^ Department of Food Science and TechnologyFederal University of TechnologyP.M.B. 704AkureOndo StateNigeria

**Keywords:** *akara*, antinutrient, *gbegiri*, leguminous grains, *moin‐moin*

## Abstract

The individualistic effect of unit operations of production, at household level, on some antinutritional factors in selected cowpea‐based food products (*moin‐moin, akara,* and *gbegiri*) was investigated. Four cowpea types (IT93K‐452‐1, IT95K‐499s‐35, IT97K‐568‐18, and market sample) were used for the study, whereas the three traditional food products were produced from each of the cowpea types, respectively. The results revealed that every unit operation involved in the production of *moin‐moin, akara or gbegiri* contributed to the overall reduction of trypsin inhibitor activity (TIA), phytic acid (PA), and tannin; though at varying degrees. In the production of *moin‐moin*, the major contributions to the overall reduction in TIA were from steaming (64.2–72.0%), second‐stage soaking (9.7–11.9%), and dehulling (9.4–10.2%). The contributions to the overall reduction in PA were from dehulling (34.0–40.4%), preliminary soaking (15.4–21.0%), and steaming (7.8–14.0%), whereas that of tannin were from dehulling (39.7–47.6%), steaming (19.6–24.7%), and preliminary soaking (9.8–15.9%). For *akara* production, the major contributions to TIA reduction were from deep frying (64.2–72.0%), second‐stage soaking (9.7–11.9%), and dehulling (9.4–10.2%). The PA reduction was from dehulling (34.0–40.4%), preliminary soaking (15.4–21.0%), and deep frying (9.6–15.9%), whereas that of tannin reduction was from dehulling (39.7–47.6%), deep frying (20.7–25.3%), and preliminary soaking (9.8–15.9%). In the production of *gbegiri*, the overall reduction in TIA was contributed from pressure cooking (79.0–84.8%), preliminary soaking (5.8–11.3%), and dehulling (9.4–10.2%). The reduction in PA was contributed by dehulling (34.0–40.4%), pressure cooking (24.7–35.0%), and preliminary soaking (15.4–21.0%), whereas the overall reduction in tannin content was similarly contributed by dehulling (39.7–47.6%), pressure cooking (29.8–34.4%), and preliminary soaking (9.8–15.9%).

## Introduction

Cowpea (*Vigna unguiculata* L.) is an important grain legume in East and West African countries (Hung et al. 1990). Indeed, it is the most widely consumed legume seeds in Nigeria (Onigbinde and Akinyele 1983). The grain legume serves as the largest single contributor to the total protein intake of many rural and urban families while efforts are continually being made to increase cowpea production as a means of providing a cheaper source of protein to the teeming consumers (Ogun et al. 1989). The benefit of low‐cost dietary proteins from the traditional cowpea‐based food products is considered enormous, particularly in the developing countries, due to the high cost and limited availability of animal proteins (Sathe and Salunkhe 1981). The chemical and nutritional compositions of cowpea, as well as its cooking properties, vary considerably according to environmental and genetic factors (Giami 2005). Many traditional food products are derivable from cowpea, particularly in Nigeria, which include *akara* (fried cowpea paste containing seasonings), *moin‐moin* (steamed cowpea paste containing seasonings), *ekuru* (steamed cowpea paste with no seasonings), *gbegiri* (cowpea soup), *ewa ibeji* (cooked and softened cowpea), etc. The processing technologies for producing these cowpea‐based traditional food products are generally at artisanal level and so efforts have been made toward the development of improved technologies for producing ready‐to‐use cowpea meal and flour specifically for use in *akara* and *moin‐moin* preparation (Ngoddy et al. 1986; Phillips and McWatters 1991).

Apart from the nutritional benefits derivable from cowpea, the grain legume also contains certain antinutritional factors which include trypsin inhibitors (Kochhar et al. 1988; Onwuka 2006; Kalpanadevi and Mohan 2013), tannin (Akinyele 1989; Ogun et al. 1989; Ghavidel and Prakash 2007), and phytic acid (Ologhobo and Fetuga 1984; Uzogara et al. 1990; Khattab and Arntfield 2009), among others. These antinutrients serve as limiting factors in the utilization of cowpea for both human and animal as they make bioavailability of certain nutrients impossible. Trypsin inhibitor is a substance that has the ability to inhibit proteolytic activity of certain enzymes especially trypsin (Liener 2001). The negative effect of tannin has to do with its interference with protein digestion by binding dietary protein into an indigestible form (Bressani et al. 1982). The phytate has been implicated to decrease the bioavailability of essential minerals (Ca, Mg, Mn, Zn, Fe, and Cu) and can also form a phytate‐protein complex thereby interfering in protein utilization (Oberleas and Harland 1981).

Various attempts have been made by researchers to increase the utilization of cowpea through a wide range of appropriate processing techniques. Wang et al. (1997) investigated the combined effects of soaking, water, and steam blanching on the antinutritional factors in cowpea. It was found that a combination of soaking and steam blanching resulted in higher reduction of trypsin inhibitor activity than a combination of soaking and water blanching. Preet and Punia (2000) studied the role of soaking, dehulling, and germination on the antinutritional content of cowpea. The finding here was that each of the treatments contributed significantly in reducing the phytic acid and polyphenol content of cowpea with dehulling being the most effective in the reduction of polyphenolic content. Onwuka (2006) assessed the effect of soaking, boiling, and combination of these treatments on the antinutritional factors in cowpea. It was found that the combination of soaking and boiling was more potent than individual soaking or boiling in the reduction of trypsin inhibitor, cyanogenic glycoside, hemagglutinin, alkaloids, and tannin.

Ghavidel and Prakash (2007) also investigated the impact of germination and dehulling on the antinutrient component of cowpea. The finding here was that phytic acid and tannin were fairly reduced by germination while the impact of dehulling was more effective. Khattab and Arntfield (2009) examined the influence of physical treatments (water soaking, boiling, roasting, microwave cooking, autoclaving, fermentation, and micronization) on the antinutritional component of cowpea. It was found that all treatments evaluated caused significant decreases in tannin, phytic acid, trypsin inhibitor activity, and oligosaccharides. However, boiling caused the highest reduction in tannin followed by autoclaving and microwave cooking, whereas autoclaving and germination were the most effective in reducing phytic acid content. All the heat treatments brought a total removal of trypsin inhibitor activity. Kalpanadevi and Mohan (2013) also studied the effect of hydration, cooking, autoclaving, germination and their combination on the reduction/elimination of antinutrients in *V. unguiculata*. It was found that hydration and germination processes were less effective in reducing trypsin inhibitor activity, whereas cooking and autoclaving of presoaked seeds were very effective for reducing the content of total free phenolics, tannin, phytic acid, hydrogen cyanide, trypsin inhibitors, and oligosaccharides.

Virtually all these investigations were focused toward the role of unit operations/processes, as might be encountered in the handling and processing of cowpea, on the reduction/removal of antinutritional factors but not targeted toward the production of specific cowpea‐based food products. Even where specific cowpea‐based food products were targeted (Akinyele 1989; Ogun et al. 1989), it was the cumulative role of the processing methods on the antinutritional factors that was evaluated rather than the individualistic impact of the unit operations of production. However, this study examined the contributions of individual unit operations of production to such cumulative effect.

The objective of this study therefore was to evaluate the sequential impact of unit operations of production, at household level, on some antinutritional factors in selected cowpea‐based food products.

## Materials and Methods

### Source of materials

Four cowpea types were used for this study. Three varieties, namely IT93K‐452‐1, IT95K‐499s‐35, and IT97K‐568‐18 were obtained from the International Institute of Tropical Agriculture (IITA), Ibadan, Nigeria; whereas the fourth one, a market sample (*Ewa Oloyin*), was obtained from Bodija market, Ibadan, Nigeria. The cowpea varieties (IT93K‐452‐1, IT95K‐499s‐35, and IT97K‐568‐18) were white‐coated, whereas the market sample (*Ewa Oloyin*) was brown‐coated.

### Production of *moin‐moin, akara*, and *gbegiri* using household methods

**Figure 1:**
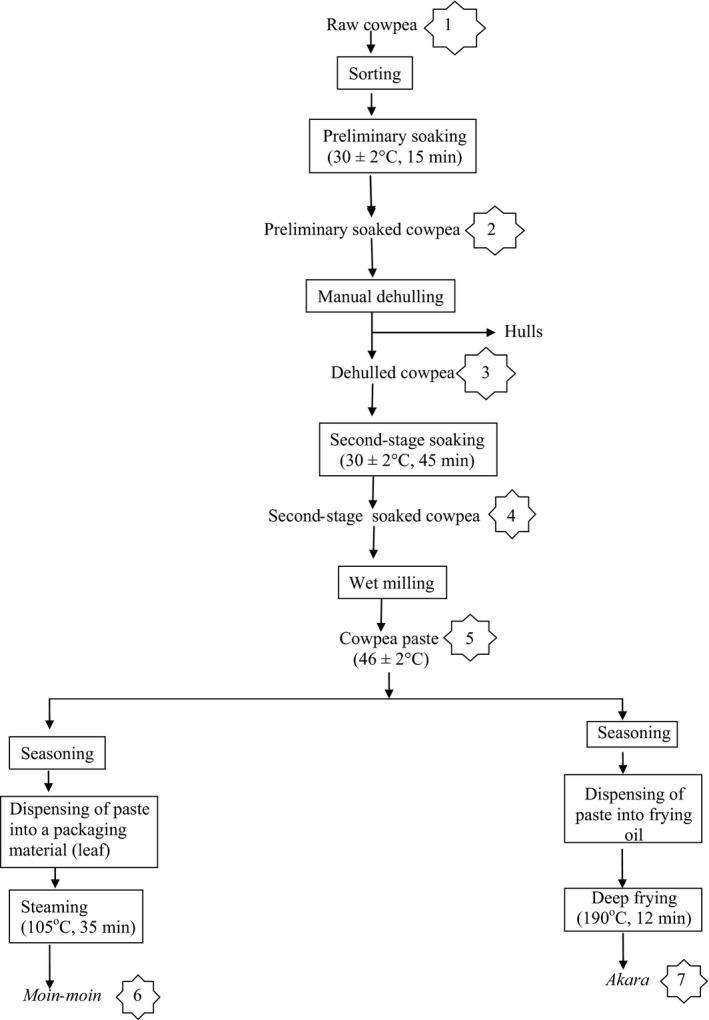
Flowchart illustrating the household production of *moin‐moin* and *akara* from cowpea. (The indicated numbers are points of sample collection for analysis).

*Moin‐moin* (steamed cowpea paste containing seasonings) and *akara* (fried cowpea paste containing seasonings) were, respectively, produced as illustrated in Figure 1. The various points where samples were taken for analysis are numbered accordingly.

**Figure 2:**
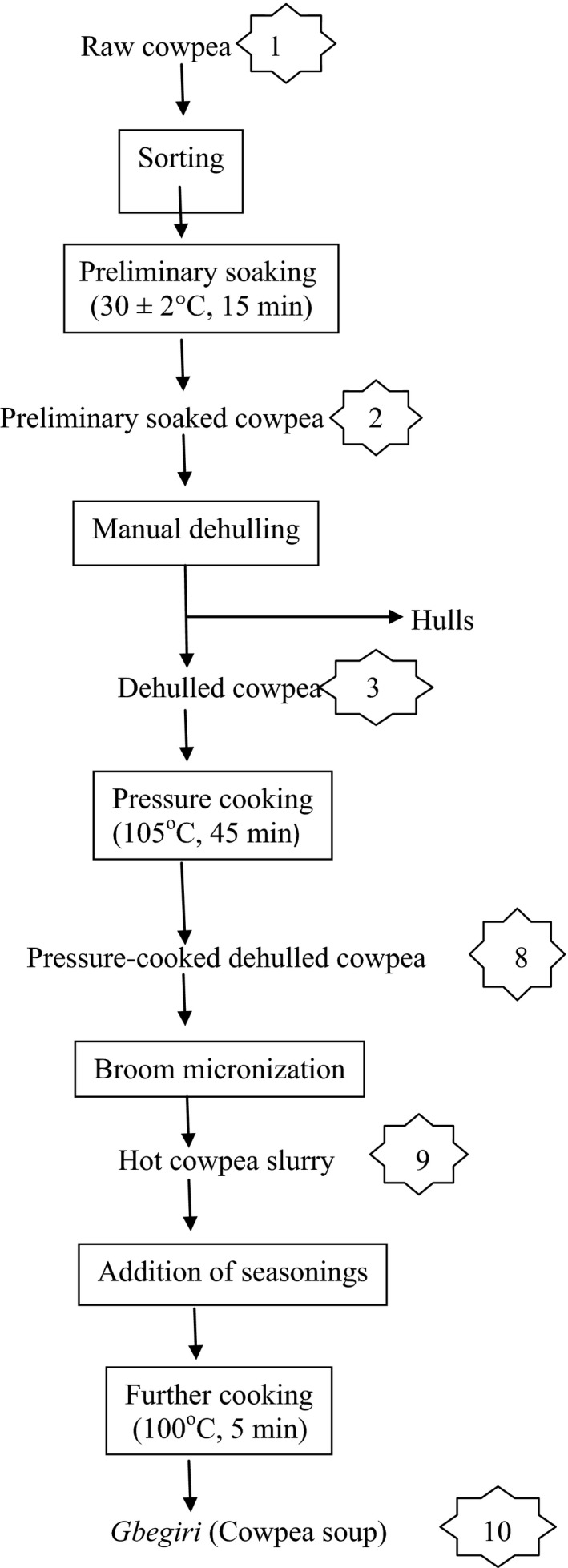
Flowchart illustrating the household production of *gbegir*i from cowpea. (The indicated numbers are points of sample collection for analysis).

*Gbegiri* (cowpea soup) was also produced as illustrated in Figure 2. The various points where samples were taken for analysis are also numbered accordingly.

Three different households were used to prepare the products from where samples were collected for analysis, whereas each cowpea variety was used to prepare the food products, respectively.

### Sample collection and preparation

Samples for the analysis were taken from 10 different points as indicated in Figures 1 and 2. Sample preparation was done following the method of Khattab and Arntfield (2009). Each sample was dried overnight using air draught oven (Model No. DHG‐910.1SA, Sanfa) at 55 ± 2°C, ground to pass through an 841‐μm screen and kept frozen in polyethylene bags until required for analyzed.

### Determination of trypsin inhibitor activity

The method of Kakade et al. (1974) was used to determine the trypsin inhibitor activity (TIA) of each sample using benzoyl‐DL‐arginine‐*p*‐nitroanilide (BAPNA) as substrate. A 4.0 g sample was treated with 40 mL of 0.05 mol/L sodium phosphate buffer, pH 7.5 and 40 mL of distilled water. The sample was agitated for 3 h using a magnetic stirrer and centrifuged at 700 g for 30 min at 15°C. Supernatant was diluted to obtain inhibition between 40 and 60% of enzyme activity. Incubation mixture consisted of 0.5 mL trypsin solution (5 mg/mL), 2 mL 2% (w/v) BAPNA, 1.0 mL sodium phosphate buffer (pH 7.5, 0.1 mol/L), 0.4 mL HCl (0.001 mol/L), and sample extract (0.1 mL). Total volume of incubation mixture was maintained at 4.0 mL. Incubation was carried out in a water bath at 37°C for 20 min after which 6.0 mL of 5% TCA (trichloroacetic acid) solution was added to stop the reaction. Blank sample was treated similarly through the entire determination. Absorbance (A) was read at 410 nm wavelength in a spectrophotometer (UV‐160A; Shimadzu, Osaka, Japan). Results were expressed as trypsin inhibitor units (TIU). One TIU was defined as an increase of 0.01 in absorbance units under conditions of assay. Trypsin inhibitory activity was defined as the number of TIU.

### Determination of phytic acid content

The method of Wheeler and Ferrel (1971) was used to determine the phytic acid content of each sample. A 2.0 g sample measurement was used for the extraction. A standard curve was prepared expressing the results as Fe(NO_3_)_3_ equivalent. Phytate phosphorus was calculated from the standard curve assuming a 4:6 iron to phosphorus molar ratio. The phytic acid content was also calculated by multiplying the amount of phytate phosphorous by the factor 3.55 based on the empirical formula C_6_P_6_O_2_H_18_ and result expressed as mg/100 g sample.

### Determination of tannin content

Tannin was determined according to the method of Price and Butler (1977) with a minor modification. Sixty milligrams (60 mg) of ground sample were shaken manually for 1 min in 3.0 mL methanol. The mixture was filtered followed by mixing the filtrate with 50 mL distilled water and analyzed within an hour. About 3.0 mL of 0.1 mol/L FeCl_3_ in 0.1 mol/L HCl were added to 1 mL filtrate, followed immediately by the addition of 3.0 mL freshly prepared K_3_Fe(CN)_6_. The absorbance was read on a spectrophotometer (Shimadzu UV‐1700, Tokyo, Japan) at 720 nm after 10 min from the addition of 3.0 mL of 0.1 M FeCl_3_ and 3.0 mL of 0.008 mol/L K_3_Fe(CN)_6_. Similar treatments were also carried on the blank. Results were expressed as tannic acid equivalent (mg/100 g sample), calculated from a calibration curve using tannic acid.

### Temperature measurement

The temperature of the environment and sample was measured using different types of thermometer (0–110°C and 0–360°C).

### Statistical analysis

All determinations reported in this study were carried out in triplicates. In each case, a mean value and standard deviation were calculated. Analysis of variance (ANOVA) was also performed and separation of the mean values was by Duncan's Multiple Range Test at *P* < 0.05 using Statistical Package for Social Scientists (SPSS) software, version 16.0 (SPSS Inc., Chicago, IL); on a personal computer.

## Results and Discussion

### Elimination of trypsin inhibitor activity in *moin‐moin, akara,* and *gbegiri* as influenced by the unit operations of production

Table 1 summarizes the effect of unit operation of production and cowpea variety on the trypsin inhibitor activity (TIA) in *moin‐moin* (steamed cowpea paste) and *akara* (fried cowpea paste), whereas Table 2 summarizes that in *gbegiri* (cowpea soup). The cowpea varieties used for preparing the food products contained different concentrations of TIA ranging between 2349.7 and 2844.2 TIU/g with significant difference (*P* ≤ 0.05). Sample IT95K‐499s‐35 and the market sample gave the lowest and highest TIA values, respectively. Genetic and environmental factors have been implicated to be responsible for variability in the composition of antinutrients in cowpea (Terryn and Montagu 2008; Carvalho et al. 2012; Owolabi et al. 2012). The contributory reduction of TIA by the preliminary soaking, as a common unit operation in the production of *moin‐moin, akara,* and *gbegiri,* respectively, ranged between 6.2 and 11.3%. It had earlier been observed that soaking plays a significant role in the reduction of trypsin inhibitor activity as the inhibitor is a low molecular weight protein (Clemente and Domoney 2006) capable of leaching during soaking.

**Table 1:** Effect of unit operation of production on the trypsin inhibitor activity (TIA) in *moin‐moin* and *akara* a

Material	Corresponding unit operation	Source of *moin‐moin* and *akara*							
		IT93K‐452‐1		IT95K‐499s‐35		IT97K‐568‐18		Market sample (*Ewa‐oloyin)*	
		Trypsin inhibitor activity (TIU/g sample)	Contributory reduction capacity^b^ (%)	Trypsin inhibitor activity (TIU/g sample)	Contributory reduction capacity (%)	Trypsin inhibitor activity (TIU/g sample)	Contributory reduction capacity (%)	Trypsin inhibitor activity (TIU/g sample)	Contributory reduction capacity (%)
Raw cowpea		2523.4 ± 13.4^c^	–	2349.7 ± 9.8^d^	–	2638.5 ± 12.6^b^	–	2844.2 ± 11.7^a^	–
Preliminary soaked cowpea	Preliminary soaking	2366.1 ± 6.2^b^	6.2	2152.4 ± 10.2^d^	8.4	2341.8 ± 8.5^c^	11.3	2679.1 ± 9.7^a^	5.8
Dehulled cowpea	Dehulling	2111.5 ± 11.6^b^	10.1	1913.8 ± 6.6^d^	10.2	2084.2 ± 7.6c	9.8	2412.3 ± 5.5^a^	9.4
Second‐stage soaked cowpea	Second‐stage soaking	1866.7 ± 5.7^b^	9.7	1633.2 ± 5.1^d^	11.9	1771.3 ± 4.5^c^	11.9	2114.6 ± 7.2^a^	10.5
Paste	Wet milling	1792.2 ± 8.3^b^	4.0	1595.6 ± 9.1^d^	1.6	1692.5 ± 8.1^c^	3.8	2047.9 ± 6.3^a^	2.3
*Moin‐moin*	Steaming	0	71.0	0	67.9	0	64.2	0	72.0
*Akara*	Deep frying	0	71.0	0	67.9	0	64.2	0	72.0
Overall reduction in trypsin inhibitor activity (%)			Moinmoin = 100 Akara = 100		Moinmoin = 100 Akara = 100		Moinmoin = 100 Akara = 100		Moinmoin = 100 Akara = 100

a Results are mean values of data from three different households ± standard deviation. Mean value within the same row having the same letter are not significantly different at *P* ≤ 0.05.

b Contributory reduction capacity (%) was calculated with respect to the initial total trypsin inhibitor activity (TIA) in the raw cowpea.

**Table 2:** Effect of unit operation of production on the trypsin inhibitor activity (TIA) in *gbegiri* a

Material	Corresponding unit operation	Source of *gbegiri*							
		IT93K‐452‐1		IT95K‐499s‐35		IT97K‐568‐18		Market sample (*Ewa‐oloyin)*	
		Trypsin inhibitor activity (TIU/g sample)	Contributory reduction capacity^b^ (%)	Trypsin inhibitor activity (TIU/g sample)	Contributory reduction capacity (%)	Trypsin inhibitor activity (TIU/g sample)	Contributory reduction capacity (%)	Trypsin inhibitor activity (TIU/g sample)	Contributory reduction capacity (%)
Raw cowpea		2523.4 ± 13.4^c^	–	2349.7 ± 9.8^d^	–	2638.5 ± 12.6^b^	–	2844.2 ± 11.7^a^	–
Preliminary soaked cowpea	Preliminary soaking	2366.1 ± 6.2^b^	6.2	2152.4 ± 10.2^d^	8.4	2341.8 ± 8.5^c^	11.3	2679.1 ± 9.7^a^	5.8
Dehulled cowpea	Dehulling	2111.5 ± 11.6^b^	10.1	1913.8 ± 6.6^d^	10.2	2084.2 ± 7.6^c^	9.8	2412.3 ± 5.5^a^	9.4
Pressure‐cooked dehulled cowpea	Pressure cooking	0	83.7	0	81.4	0	79.0	0	84.8
Hot cowpea slurry	Broom micronization	0	0	0	0	0	0	0	0
*Gbegiri*	Further cooking	0	0	0	0	0	0	0	0
Overall reduction in trypsin inhibitor activity of *gbegiri* (%)			100		100		100		100

a Results are mean values of data from three different households ± standard deviation. Mean value within the same row having the same letter are not significantly different at *P* ≤ 0.05.

b Contributory reduction capacity (%) was calculated with respect to the initial total trypsin inhibitor activity (TIA) in the raw cowpea.

The contributory reduction capacity of dehulling with respect to TIA ranged between 9.4 and 10.2%. The lowest and highest reduction capacities were observed in the market sample and IT95K‐499s‐35, respectively. The implication of this observation is that a modest amount of TIA is present in the seed coat while the variations in the contributory reduction of TIA, through dehulling, in the cowpea varieties are indications that varietal differences do play a role in TIA reduction (Ologhobo and Fetuga 1984). The second‐stage soaking contributed between 9.7 and 11.9% to the overall reduction of TIA in the course of *moin‐moin* and *akara* production, respectively. The lowest reduction capacity was observed in IT93K‐452‐1, whereas the highest reduction capacity was observed in both IT95K‐499s‐35 and IT97K‐568‐18. The reduction in TIA during the second‐stage soaking was higher than that of the preliminary soaking in all the cowpea varieties and this observation may be attributed to the elongated second‐stage soaking period (45 min) which might have allowed greater leaching of trypsin inhibitor into the soaking water. In addition, the absence of the seed coat (hulls) during the second‐stage soaking period might have allowed trypsin inhibitor to leach more easily.

The wet milling of cowpea to obtain the paste contributed between 1.6 and 4.0% to the overall reduction of TIA in the production of *moin‐moin* and *akara,* respectively. This modest contributory reduction in the TIA may be attributed to an enlarged surface area of the paste with higher temperature (46 ± 2°C). The steaming (*moin‐moin*) and deep frying (*akara*) operations contributed between 64.2 and 72.0% for both products. This is the highest contributory reduction in TIA for these two food products as total elimination was observed. Both IT97K‐568‐18 and the market sample exhibited the lowest and highest reduction capacities, respectively, for the TIA. It had earlier been observed that high‐temperature processing operations such as boiling, steaming, microwave cooking, roasting, or autoclaving are capable of causing total elimination of TIA (Burns 1986; Liener 1986; Vidal‐Valverde et al. 1994).

In the case of *gbegiri* production (Table 2), the TIA was equally totally eliminated by the pressure cooking giving a contributory reduction capacity of between 79.0 and 84.8%. Both IT97K‐568‐18 and the market sample exhibited the lowest and highest reduction capacities, respectively. The seeming higher contributory reduction capacity of pressure cooking for TIA than that of steaming and deep frying may be attributed to the higher TIA concentration in the dehulled cowpea prior to the heat treatment.

The two subsequent unit operations (broom micronization and further cooking) involved in *gbegiri* production did not contribute in any way to TIA elimination in the food product. Broom micronization is essentially a unit operation in *gbegiri* production which involves the use of a short‐length broom to manually beat already cooked and softened cowpea in order to obtain free flow cowpea slurry. The zero‐level TIA found in *gbegiri* as observed in this study seems to contradict the findings of a previous worker (Akinyele 1989) who reported a residual TIA in *gbegiri*.

### Effect of unit operations of production of *moin‐moin, akara,* and *gbegiri* on the phytic acid

The effect of unit operations of production on the phytic acid (PA) content of *moin‐moin* and *akara* is shown in Table 3 whereas that of *gbegiri* is in Table 4. There were variations in the PA content of the raw cowpea ranging between 680.5 and 984.3 mg/100 g with significant difference (*P* ≤ 0.05). Both IT97K‐568‐18 and the market sample had the lowest and highest PA values, respectively. The preliminary soaking was found to contribute between 15.4 and 21.0% to the overall PA reduction in the production of *moin‐moin, akara,* and *gbegiri*. Both IT93K‐452‐1 and IT95K‐499s‐35 exhibited the lowest and highest reduction capacities for the PA, respectively. Soaking had earlier been observed to be capable of reducing PA content in cowpea due to its leaching tendency into the soaking water (Preet and Punia 2000; Onwuka 2006) in addition to the hydrolytic tendency of PA by the endogenous phytase (Reddy and Pierson 1994; Sandberg and Andlid 2002).

**Table 3:** Effect of unit operation of production on the phytic acid (PA) content in *moin‐moin* and *akara* a

Material	Corresponding unit operation	Source of *moin‐moin* and *akara*							
		IT93K‐452‐1		IT95K‐499s‐35		IT97K‐568‐18		Market sample (*Ewa‐oloyin*)	
		Phytic acid content (mg/100 g sample)	Contributory reduction capacity^b^ (%)	Phytic acid content (mg/100 g sample)	Contributory reduction capacity (%)	Phytic acid content (mg/100 g sample)	Contributory reduction capacity (%)	Phytic acid content (mg/100 g sample)	Contributory reduction capacity (%)
Raw cowpea		723.9 ± 5.6^c^	–	784.2 ± 6.8^b^	–	680.5 ± 7.1^d^	–	984.3 ± 6.3^a^	–
Preliminary soaked cowpea	Preliminary soaking	612.4 ± 4.2^b^	15.4	619.6 ± 4.4^b^	21.0	551.8 ± 5.5^c^	18.9	792.4 ± 7.1^a^	19.5
Dehulled cowpea	Dehulling	358.2 ± 6.8^b^	35.0	302.8 ± 6.1^c^	40.4	307.2 ± 4.9^c^	35.9	458.2 ± 4.8^a^	34.0
Second‐stage soaked cowpea	Second‐stage soaking	292.4 ± 3.7^b^	9.1	238.3 ± 3.5^d^	8.2	253.4 ± 3.7^c^	7.9	392.3 ± 5.7^a^	6.7
Paste	Wet milling	204.8 ± 4.2^b^	12.1	163.7 ± 3.1^d^	9.5	184.7 ± 4.6^c^	10.1	304.9 ± 4.1^a^	8.9
*Moin‐moin*	Steaming	134.7 ± 2.5^b^	9.7	102.4 ± 2.8^c^	7.8	93.6 ± 2.5^d^	13.4	166.7 ± 3.4^a^	14.0
*Akara*	Deep frying	111.3 ± 2.9^b^	12.9	88.1 ± 2.2^c^	9.6	77.9 ± 2.2^d^	15.7	148.2 ± 2.8^a^	15.9
Overall reduction in phytic acid content (%)			Moinmoin = 81.3 Akara = 84.5		Moinmoin = 86.9 Akara = 88.7		Moinmoin = 86.2 Akara = 88.5		Moinmoin = 83.1 Akara = 85.0

a Results are mean values of data from three different households ± standard deviation. Mean value within the same row having the same letter are not significantly different at *P* ≤ 0.05.

b Contributory reduction capacity (%) was calculated with respect to the initial phytic acid (PA) in the raw cowpea.

**Table 4:** Effect of unit operation of production on the phytic acid (PA) content in *gbegiri* a

Material	Corresponding unit operation	Source of *gbegiri*							
		IT93K‐452‐1		IT95K‐499s‐35		IT97K‐568‐18		Market sample (Ewa‐oloyin)	
		Phytic acid content (mg/100 g sample)	Contributory reduction capacity^b^ (%)	Phytic acid content (mg/100 g sample)	Contributory reduction capacity (%)	Phytic acid content (mg/100 g sample)	Contributory reduction capacity (%)	Phytic acid content (mg/100 g sample)	Contributory reduction capacity (%)
Raw cowpea	–	723.9 ± 5.6^c^	–	784.2 ± 6.8^b^	–	680.5 ± 7.1^d^	–	984.3 ± 6.3^a^	–
Preliminary soaked cowpea	Preliminary soaking	612.4 ± 4.2^b^	15.4	619.6 ± 4.4^b^	21.0	551.8 ± 5.5^c^	18.9	792.4 ± 7.1^a^	19.5
Dehulled cowpea	Dehulling	358.2 ± 6.8^b^	35.0	302.8 ± 6.1^c^	40.4	307.2 ± 4.9^c^	35.9	458.2 ± 4.8^a^	34.0
Pressure‐cooked dehulled cowpea	Pressure cooking	112.9 ± 3.1^c^	33.9	109.4 ± 5.2^c^	24.7	123.7 ± 3.3^b^	27.0	203.7 ± 4.4^a^	25.9
Hot cowpea slurry	Broom micronization	103.4 ± 2.5^bc^	1.3	98.5 ± 4.1^c^	1.4	108.6 ± 4.1^b^	2.2	184.9 ± 5.2^a^	1.9
*Gbegiri*	Further cooking	42.8 ± 2.2^c^	8.4	45.7 ± 2.3^c^	6.7	52.9 ± 2.7^b^	8.2	63.4 ± 3.2^a^	12.3
Overall reduction in phytic acid content of *gbegiri* (%)			94.1		94.2		92.2		93.6

a Results are mean values of data from three different households ± standard deviation. Mean value within the same row having the same letter are not significantly different at *P* ≤ 0.05.

b Contributory reduction capacity (%) was calculated with respect to the initial phytic acid (PA) in the raw cowpea.

The dehulling operation contributed between 34.0 and 40.4% to the overall reduction of PA in the production of *moin‐moin, akara,* and *gbegiri,* respectively. The lowest and highest reduction capacities for PA were exhibited by the market sample and IT95K‐499s‐35, respectively. Most PA had been observed to be present in the outer aleurone layers of leguminous seeds (Deshpande et al. 1982) with the implication that dehulling could substantially remove it, hence the relative high PA reduction as observed in this study.

The contributory reduction capacity of the second‐stage soaking to the overall PA reduction ranged between 6.7 and 9.1%. The reduction levels of PA by the second‐stage soaking were generally observed to be lower than that of the preliminary soaking in spite of the longer soaking period. This observation may be due to the previous removal of the hulls which, most probably, had contributed to the reduced leaching of PA into the soaking water. The wet milling of the dehulled cowpea contributed between 8.9 and 12.1% to the overall PA reduction in the course of *moin‐moin* and *akara* production, respectively. Wet milling essentially resulted in paste with a larger surface area and higher temperature (46 ± 2°C) which could facilitate an effective enzyme‐substrate interaction. Grenier and Konietzny (1999) had earlier observed that the optimal temperatures for the intrinsic plant phytases were between 45°C and 65°C. This might have influenced the modest contribution of wet milling to the overall reduction of the PA.

The steaming operation in *moin‐moin* production was observed to contribute between 7.8 and 14.0% to the overall PA reduction, whereas deep frying in *akara* production exhibited a contributory reduction capacity of between 9.6 and 15.9%. Both IT95K‐499s‐35 and the market sample contributed the lowest and highest reduction levels, respectively, for both products. Heat treatment generally had been observed to reduce PA concentration partly due to the heat‐labile nature of the acid coupled with possible formation of insoluble complexes such as calcium and magnesium phytates (Crean and Haisman 1963; Udensi et al. 2007). Nevertheless, the contributory reduction capacity of deep frying to the overall PA reduction was generally observed to be higher than that of steaming due to its higher temperature (190°C) as against that of 105°C for steaming. The overall reduction in PA content in *moin‐moin* was observed to range between 81.3 and 86.9%, whereas that in *akara* was between 84.5 and 88.7%. This implies that the two food products, as consumed, could still contain residual PA.

In the production of *gbegiri* (Table 4), the contributory reduction capacity of pressure cooking in the overall reduction of PA was between 24.7 and 33.9%. Broom micronization, as a unit operation of production, accounted for 1.3–2.2% reduction capacity level in the overall PA reduction, whereas further cooking also accounted for 6.7–12.3%. The contributory reduction capacities of pressure cooking and further cooking may be attributed to thermal destruction, whereas that of broom micronization may be attributed to surface area enlargement which might have facilitated somewhat mechanical destruction. The overall reduction in PA content in *gbegiri* was observed to range between 92.2 and 94.2%; which implies that *gbegiri*, as consumed, could still contain residual PA.

### Reduction of tannin content in *moin‐moin, akara,* and *gbegiri* as influenced by the unit operations of production

The effect of unit operation of production on the tannin content of *moin‐moin* and *akara* is presented in Table 5, whereas that of *gbegiri* is in Table 6. The concentration of tannin in the raw cowpea ranged between 2009.3 and 2411.4 mg/100 g with significant difference (*P* ≤ 0.05). Both IT95K‐499s‐35 and the market sample gave the lowest and highest values, respectively. The preliminary soaking, as a common unit operation of production for *moin‐moin, akara,* and *gbegiri*, exhibited a contributory reduction capacity of between 9.8 and 15.9%. Samples IT93K‐452‐1 and IT95K‐499s‐35 gave the lowest and highest reduction levels, respectively. The tannin reduction through preliminary soaking may be attributed to water solubility property of tannin (Kumar et al. 1979) which predisposed it toward solubilizing into the soaking water.

**Table 5:** Effect of unit operation of production on the tannin content in *moin‐moin* and *akara* a

Material	Corresponding unit operation	Source of *moin‐moin* and *akara*							
		IT93K‐452‐1		IT95K‐499s‐35		IT97K‐568‐18		Market sample (*Ewa‐oloyin*)	
		Tannin content (mg/100 g sample)	Contributory reduction capacity^b^ (%)	Tannin content (mg/100 g sample)	Contributory reduction capacity (%)	Tannin content (mg/100 g sample)	Contributory reduction capacity (%)	Tannin content (mg/100 g sample)	Contributory reduction capacity (%)
Raw cowpea	–	2219.4 ± 13.1^c^	–	2009.3 ± 10.5^d^	–	2342.8 ± 14.3^b^	–	2411.4 ± 12.7^a^	–
Preliminary soaked cowpea	Preliminary soaking	1866.2 ± 8.6^c^	15.9	1812.7 ± 11.2^d^	9.8	2053.7 ± 12.6^b^	12.3	2114.8 ± 11.2^a^	12.3
Dehulled cowpea	Dehulling	984.7 ± 6.4^a^	39.7	884.5 ± 7.8^c^	46.2	980.3 ± 7.8^ab^	45.8	968.2 ± 9.8^b^	47.6
Second‐stage soaked cowpea	Second‐stage soaking	804.5 ± 8.2^b^	8.1	780.3 ± 5.5^c^	5.2	788.9 ± 6.3^c^	8.2	857.3 ± 6.1^a^	4.6
Paste	Wet milling	513.6 ± 4.4^c^	13.1	562.8 ± 3.2^b^	10.8	567.4 ± 6.9^b^	9.5	624.7 ± 7.3^a^	9.7
*Moin‐moin*	Steaming	78.2 ± 3.1^a^	19.6	66.9 ± 2.9^bc^	24.7	71.7 ± 4.8^ab^	21.2	62.4 ± 3.9^c^	23.3
*Akara*	Deep frying	53.4 ± 2.5^a^	20.7	54.8 ± 3.6^a^	25.3	49.3 ± 3.2^a^	22.1	41.5 ± 2.8^b^	24.2
Overall reduction in tannin content (%)			Moinmoin = 96.4 Akara = 97.5		Moin‐moin = 96.7 Akara = 97.3		Moin‐moin = 97.0 Akara = 97.9		Moin‐moin = 97.5 Akara = 98.4

a Results are mean values of data from three different households ± standard deviation. Mean value within the same row having the same letter are not significantly different at *P* ≤ 0.05.

b Contributory reduction capacity (%) was calculated with respect to the initial tannin content in the raw cowpea.

**Table 6:** Effect of unit operation of production on the tannin content in *gbegiri* a

Material	Corresponding unit operation	Source of *gbegiri*							
		IT93K‐452‐1		IT95K‐499s‐35		IT97K‐568‐18		Market sample (Ewa‐oloyin)	
		Tannin content (mg/100 g sample)	Contributory reduction capacity^b^ (%)	Tannin content (mg/100 g sample)	Contributory reduction capacity (%)	Tannin content (mg/100 g sample)	Contributory reduction capacity (%)	Tannin content (mg/100 g sample)	Contributory reduction capacity (%)
Raw cowpea	–	2219.4 ± 13.1^c^	–	2009.3 ± 10.5^d^	–	2342.8 ± 14.3^b^	–	2411.4 ± 12.7^a^	–
Preliminary soaked cowpea	Preliminary soaking	1866.2 ± 8.6^c^	15.9	1812.7 ± 11.2^d^	9.8	2053.7 ± 12.6^b^	12.3	2114.8 ± 11.2^a^	12.3
Dehulled cowpea	Dehulling	984.7 ± 6.4^a^	39.7	884.5 ± 7.8^c^	46.2	980.3 ± 7.8^ab^	45.8	968.2 ± 9.8^b^	47.6
Pressure‐cooked dehulled cowpea	Pressure cooking	221.9 ± 3.5^c^	34.4	243.8 ± 4.1^b^	31.9	259.2 ± 5.2^a^	30.8	248.7 ± 6.3^b^	29.8
Hot cowpea slurry	Broom micronization	202.5 ± 3.8^c^	0.9	230.3 ± 4.5^ab^	0.7	237.7 ± 3.9^a^	0.9	229.2 ± 4.7^b^	0.8
*Gbegiri*	Further cooking	62.4 ± 2.3^d^	6.3	71.6 ± 3.2^c^	7.9	80.5 ± 2.8^b^	6.7	86.6 ± 2.6^a^	5.9
Overall reduction in tannin content of *gbegiri* (%)			97.2		96.5		96.5		96.4

a Results are mean values of data from three different households ± standard deviation. Mean value within the same row having the same letter are not significantly different at *P* ≤ 0.05.

b Contributory reduction capacity (%) was calculated with respect to the initial tannin content in the raw cowpea.

The contributory reduction capacity of dehulling in the overall tannin reduction during *moin‐moin, akara,* and *gbegiri* production was observed to range between 39.7 and 47.6%. IT93K‐452‐1 and the market sample gave the lowest and highest reduction levels, respectively. It had earlier been observed that tannin is predominantly located in the leguminous seed coats (Reddy and Pierson 1994) and therefore dehulling has the capability of its substantial removal. The second‐stage soaking contributed between 4.6 and 8.2% to the overall tannin reduction; lower than that of the preliminary soaking. This may be due to the initial removal of the hulls before soaking which, most probably, had contributed to a reduced solubilization of tannin into the soaking water.

The contribution of wet milling, as a unit operation, to the overall reduction of tannin during *moin‐moin* and *akara* production ranged between 9.5 and 13.1%. IT97K‐568‐18 and IT93K‐452‐1 gave the lowest and highest reduction levels, respectively. During wet milling, paste is formed and the temperature of the paste usually increases to 46 ± 2°C, whereas the overall surface area of the paste is enlarged. All these factors could facilitate an efficient enzyme‐substrate interaction in the paste thereby leading to tannin reduction. It had earlier been observed that tannin could be oxidized by polyphenol oxidase (endogenous enzyme) which might lead to reduction in its concentration (Reddy and Pierson 1994).

The contributory reduction capacity of steaming in the overall tannin reduction ranged between 19.6 and 24.7%. However, the contributory reduction capacity of deep frying ranged between 20.7 and 25.3%. The high temperature of steaming (105°C) and deep frying (190°C) may be implicated for this modest tannin reduction. It had earlier been observed that tannin could be degraded upon heat treatment such as boiling, roasting, and microwave cooking (Rakic et al. 2007; Udensi et al. 2007).

In the production of *gbegiri* (Table 6), the contributory reduction capacity of pressure cooking for tannin ranged between 29.8 and 34.4%. The market sample and IT93K‐452‐1 contributed the lowest and highest reduction levels, respectively. Broom micronization accounted for 0.7–0.9% reduction levels in tannin while further cooking also accounted for 5.9–7.9% reduction level. Pressure cooking and further cooking had the capacity for thermal destruction of tannin, whereas broom micronization might have caused somewhat marginal mechanical destruction. Nevertheless, the overall reduction of tannin in *moin‐moin* (96.4–97.5%), *akara* (97.3–98.4%), and *gbegiri* (96.4–97.2%); all indicated that the food products, as consumed, could still contain residual tannin content. This observation seems to contradict the findings of previous workers (Ogun et al. 1989) who reported zero‐level tannin in *moin‐moin*.

## Conclusion

The conclusion that can be drawn from this study is that the various unit operations through which cowpea is processed, in the course of preparing *moin‐moin, akara,* and *gbegiri*, contributed individually to the overall reduction in TIA, PA, and tannin. In the preparation of *moin‐moin*, the highest contributory reduction capacity for TIA was obtained from the steaming operation (64.2–72.0%); dehulling and preliminary soaking operations exhibited contributions of 34.0–40.4% and 15.4–21.0% for PA, respectively, whereas dehulling and steaming operations showed contributions of 39.7–47.6% and 19.6–24.7% for tannin, respectively.

For *akara* production, the deep frying operation had the highest reduction capacity (64.2–72.0%) for TIA; dehulling and preliminary soaking operations similarly showed contributions of 34.0–40.4% and 15.4–21.05 for PA, respectively, whereas dehulling and deep frying operations revealed contributions of 39.7–47.6% and 20.7–25.3% for tannin, respectively.

In the production of *gbegiri*, it was the pressure cooking that exhibited the highest contributory reduction capacity (79.0–84.8%) for TIA, whereas dehulling and pressure cooking operations exhibited contributions of 34.0–40.4% and 24.7–33.9% for PA as well as contributions of 39.7–47.6% and 29.8–34.4% for tannin, respectively.

## Conflict of Interest

None declared.
